# Developing 21^st ^century accreditation standards for teaching hospitals: the Taiwan experience

**DOI:** 10.1186/1472-6963-9-232

**Published:** 2009-12-15

**Authors:** Chung-I Huang, Cathy Wung, Che-Ming Yang

**Affiliations:** 1 Taipei Medical University Hospital, Taipei, Taiwan; 2 Taiwan Joint Commission on Hospital Accreditation and Quality Improvement, Taipei, Taiwan; 3 Taipei Medical University School of Health Care Administration, Taipei, Taiwan; 4 Taipei Medical University-Shuang Ho Hospital, Taipei, Taiwan

## Abstract

**Background:**

The purpose of this study is to establish teaching hospital accreditation standards anew with the hope that Taiwan's teaching hospitals can live up to the expectations of our society and ensure quality teaching.

**Methods:**

The development process lasted two years, 2005-2006, and was separated into three stages. The first stage centered on leadership meetings and consensus building, the second on drafting the new standards with expert focus groups, and the third on a pilot study and subsequent revision.

**Results:**

Our new teaching hospital accreditation standards have six categories and 95 standards as follows: educational resources (20 items), teaching and training plans and outcomes (42 items), research and results (9 items), development of clinical faculty and continuing education (8 items), academic exchanges and community education (8 items), and administration (8 items).

**Conclusions:**

The new standards have proven feasible and posed reasonable challenges in the pilot study. We hope the new standards will strengthen teaching and research, and improve the quality of hospital services at the same time.

## Background

It has been well established that not only does hospital accreditation elevate health care quality, it also improves the abilities of health care personnel [1]. It has likewise been widely accepted that hospital accreditation is paramount to patient safety [2]. Health care is a continuous process. Therefore, we cannot only survey structures for the purpose of accreditation, but also need to evaluate outcomes and processes [3]. Accreditation standards must be predetermined, agreed upon and made public [4].

Taiwan started accrediting hospitals in 1986 when the Medical Care Act aimed at improving health care quality through regulatory reform was first enacted. Accreditation was mandatory for hospitals and initially free. At first, the Department of Health (DOH), Taiwan, conducted the accreditations by itself. After the establishment of the National Health Insurance (NHI) program in 1995, only accredited hospitals were qualified to be NHI health care providers, offering strong incentives for hospitals to seek high scores for accreditation. With time, the scope of accreditation has been expanded and elaborated, requiring 12 categories of surveyors to conduct a simultaneous on-site survey of a variety of hospital services, such as internal medicine, surgery, nursing, pharmacy, radiology, laboratory medicine, psychiatry, teaching, and administration. The total number of accredited hospitals had reached 500, and over 100 of them were teaching hospitals. The criticism the old system encountered most frequently was the standards were too structure-oriented and did not put enough emphasis on process and outcomes. Moreover, hospitals also complained that too many surveyors on site at one time had interrupted the daily operation of the hospitals. In short, the scale of accreditation grew too big for the DOH to handle with its own staff, and too burdensome for the hospitals receiving on-site visits. In 1999, the DOH delegated this duty to another organization and reform was needed in response to the health care community's critique.

The Taiwan Joint Commission on Hospital Accreditation (TJCHA) is a foundation jointly endowed by the DOH and a number of health care societies [5]. TJCHA has been charged with conducting accreditation by the DOH since 1999. Hospital accreditation is still mandatory but no longer free. From its inception, the TJCHA thus faced the challenge of revising accreditation processes and standards. It first sought to condense the 12 category approach into a four category one, i.e., medicine, nursing, management and teaching. The advantages of this approach include: first, it discourages pressure to add more specialty standards due to the demand of interest groups, and secondly, it becomes more manageable to maintain a group of qualified surveyors so as to ensure the consistency of survey results. In line with this new approach, the standards have to be rewritten and reorganized. In addition, the new standards also need to focus less on structure, instead more on process and outcome.

Teaching hospitals are defined, according to the Medical Care Act of Taiwan, as hospitals with teaching, researching and training facilities for the purposes of training physicians, paramedics, and medical and paramedical students that have passed the teaching hospital accreditation [6]. Although the number of accreditation standards with respect to teaching increased over the years, the old standards were structure-oriented and there was still no substantive standard with respect to training processes and outcomes. For instance, we have two levels of teaching hospitals, A and B. A level hospitals tend to provide tertiary care and are larger hospitals in terms of bed numbers, with over 500 acute care beds. In contrast, B level hospitals are more likely to be smaller community hospitals. Until 2004, the basic requirements for both levels in the standards included the number of qualified teaching physicians, teaching equipment, library, number of teaching activities, the hours of bedside teaching, the number of publications, and the amount of money spent on teaching, training and research, etc [7].

Although there is no identical teaching hospital accreditation system internationally to what we have in Taiwan, in the sense that we have bundled hospital accreditation and teaching hospital accreditation together, there are still other examples that we can reference in revising our standards. For instance, the World Federation for Medical Education (WFME) has established global standards for quality in basic medical education, and in postgraduate medical education. The WFME basic medical education quality improvement standards look at mission and objectives, educational program, students, student assessment, academic staff/faculty, educational resources, program evaluation, governance and administration, and continuous renewal [8]. Similarly, WFME postgraduate medical education quality improvement standards focus on mission and outcomes, training process, assessment of trainees, trainees, staffing, training settings and educational resources, evaluation of training process, governance and administration, and continuous renewal [9].

The Accreditation Council for Graduate Medical Education (ACGME) of the United States has been accrediting resident training programs for many years. ACGME has established institutional and common program requirements to regulate all resident training institutions and programs. The common program requirements in particular evaluate program personnel and resources, resident appointments, program curricula, resident duty hours and working environment, evaluation, experimentation and innovation [10]. The institutional requirements stress organization and responsibilities, responsibilities for residents, the graduate medical education committee, and internal review [11].

The purpose of this study is to establish teaching hospital accreditation standards anew with the hope that Taiwan's teaching hospitals can live up to the expectations of our society and ensure quality teaching. All in all, we hope establishing a better teaching hospital accreditation system can help cultivate excellent physicians and other health care professionals so as to elevate health care quality.

## Methods

The development process lasted for two years, 2005-2006, and was separated into three stages. The first stage centered on leadership meetings and consensus building, the second on drafting the new standards with an expert focus groups, and the third on a pilot study and subsequent revision. This reform was mandated and approved by the DOH to be executed by the TJCHA, and no human subjects were involved in the process.

### First stage

Three leadership meetings were conducted at this stage to gather the opinions of leading health care professionals. The attendees, 128 in total, included the members of the medical education committee of the Ministry of Education (MOE), officers from the DOH, representatives from teaching hospitals, accreditation surveyors, deans of medical schools, etc. The consensus reached at this stage included the following:

1. The teaching survey team for hospital accreditation should survey both medical and paramedical education.

2. The weighting of research and teaching respectively should be adjusted.

3. The survey results should emphasize qualitative description rather than purely quantitative scores.

4. Surveyors should be able to review trainees during accreditation to evaluate how much they benefit from their training.

5. Training should also be community health-oriented.

6. Health care quality and efficacy should be the targets of evaluation.

### Second stage

This stage was primarily conducted via a focus group method. Fifteen experts were invited to form the focus group. All these experts had experience as surveyors and came from various hospitals and the medical education committee of MOE. These experts were further assigned to three task forces: teaching and research; clinical faculty development, budgeting and administration; and the roles and function of teaching hospitals in communities.

In addition to the separate efforts of each task force, 16 focus group meetings were conducted in order to come up with the new teaching hospital accreditation standards draft. The initial draft encompassed six categories and 96 items including: educational resources (20 items), teaching and training plans and outcomes (43 items), research and results (9 items), development of faculty and continuing education (8 items), academic exchanges and the roles and function of the hospital in the community (8 items), and administration (8 items).

### Third stage

In order to ascertain the appropriateness and applicability of the new standards, a field pilot study, consisting of pilot surveys, was carried out. Feedback gathered from the pilot study aimed at improving the standards. Twenty four hospitals volunteered and yet only 11 were selected by random stratified sampling.

Fifty one surveyors participated in the field test. TJCHA does not employ professional surveyors; that is, no surveyor is its full time employee. All of the surveyors are volunteer health care professionals. In practice, TJCHA will select qualified volunteers, give them proper training and send them out for site visits in their own spare time. Health care professionals are willing to volunteer out of altruism and also because of being a surveyor conveys prestige. For the purpose of this pilot test, TJCHA chose the most experienced surveyors from their pool of volunteers. All of them attended the pre-survey consensus meetings.

For on-site surveys of hospitals that train both resident doctors and medical students, four surveyors were dispatched including three for medical education and one for paramedical education; whereas for institutions training only resident doctors, three surveyors were sent, including two for medical education and one for paramedical education. The duration of each survey varies according to hospital size, i.e., 2.5 days for those with over 500 beds, two days for 250-499 beds, and one day for 100-249 beds. Feedback was collected from the surveyors and the participating hospitals through meetings and questionnaires. Afterwards, the focus group of experts was reconvened to modify the standards.

## Results

Eleven hospitals participated in the pilot study selected through stratified random sampling of the volunteers. There were three level A and eight level B teaching hospitals. The level A teaching hospitals are all tertiary care medical centers. Of the roughly 100 teaching hospitals, about 20 were level A teaching hospitals. Geographical distribution was taken into account; the three medical centers were in northern, central and southern Taiwan. As to the level B teaching hospitals, three are from the northern region, three from the center and two from the south.

Pass and fail is assessed on a standard-by-standard basis. The overall pass rate was 89.1%. Investigators then looked at the pass rate for each category and each standard (Table 1). For category one--educational resources--the pass rate was 91.8% and for 12 of 20 items, 100% passing was achieved. The lowest pass rate was 45.5% for standard 1.1.5: All training plans of resident doctors and medical students should have a qualified program director.

**Table 1:** The pass-fail results of the pilot study for each category of standards (N = 11)

Category	Pass		Fail	
	
	numbers	%	numbers	%
Educational resources (20 items)	202	91.8	18	8.2

Teaching and training plans and their outcomes (43 items)	433	91.6	40	8.5

Research and results (9 items)	78	78.8	21	21.2

Development of clinical faculty and continuing education (8 items)	74	84.1	14	15.9

Academic exchanges and community education (8 items)	83	94.4	5	5.6

Administration (8 items)	71	80.7	17	19.3

Total (96 items)	941	89.1	115	10.9

For category two teaching and training plans and outcomes, the pass rate was 91.6% and 100% passing was achieved for 21 standards (out of 42 items). The lowest pass rate was 54.6% for standard 2.2.11: The average overall pass rate for internal medicine, surgery, obstetrics and gynecology, and pediatrics board exams for the past three years was above 80%.

For category three, research and results, the pass rate was 78.8% and 100% passing was achieved for four standards (out of nine items). This category had the lowest overall pass rate of the six categories. The lowest pass rate was 54.6% for two standards: 3.2.2 stipulating that physicians should have good research results, and 3.2.3 stating that the execution and results of paramedicals' research must be good.

For category four, development of faculty and continuing education, the pass rate was 84.1% and 100% passing was achieved for three standards (of eight items). The lowest pass rate was 63.6% for two standards: 4.1.1, requiring a faculty development system for physicians, which is effectively operational, and 4.1.4, teaching incentives for paramedicals should be clearly promulgated and executed fully so as to encourage dedication to teaching activities.

For category 5, academic exchanges and the roles and function of the hospital in the community, the pass rate was 94.4% and 100% passing was achieved for six standards (out of eight items). This category had the highest overall pass rate of the six categories. The lowest pass rate, 63.6%, was for standard 5.2.2: participating in international medical aid and disaster relief and assisting in establishing systems and personnel training.

For category 6, administration, the pass rate was 80.7% and 100% passing was achieved for only one of eight standard items. The lowest pass rate was 54.6% for standard 6.3.1, mandating separate funding for teaching, research and advanced study, which could be supported by budgeting and final accounting data, and appropriate proportionate allocation (including physicians and paramedicals).

After post-test revision, although there are still 6 categories, some of the category titles and 16 standards were modified. There are finally 95 remaining standards (Table 2). The whole set therefore consists of 6 categories and 95 standards. The final version includes specifically educational resources (20 items), teaching and training plans and outcomes (42 items), research and results (9 items), development of clinical faculty and continuing education (8 items), academic exchanges and community education (8 items), and administration (8 items).

**Table 2:** Statistics on changes made to the standards

Category	Initial numbers of standards	Final numbers of standards	Numbers of standards revised	
			
			number	%
Educational resources	20	20	6	30.0

Teaching and training plans and outcomes	43	42	7	16.3

Research and results	9	9	0	0

Development of clinical faculty and continuing education	8	8	0	0

Academic exchanges and community education	8	8	2	25.0

Administration	8	8	1	12.5

Total	96	95	16	16.6

## Discussion

Our current system is compatible with the trend that hospital accreditation is operated by a non-profit organization for the whole nation and linked to health care reimbursement [12]. Similar examples can be found around the globe, such as the Joint Commission [13], the Japan Council for Quality Health Care (JCQHC) [14], Accreditation Canada [15], and the Australian Council on Health care Standards (ACHS) [16]. All these systems have provided some guidance for revising our teaching accreditation standards.

Although there is no identical teaching hospital accreditation system elsewhere in the world, we still looked to the systems of Japan, Zambia, Australia, and other places for references [2,16,17]. Based on our own experience, in conjunction with the global trend, the new Taiwanese system focuses more on process and outcomes and has the following features.

Category 1, educational resources, includes standards for faculty, equipment, libraries, and training facilities. These are basic standards and appear to be mostly structure oriented. However, we designed them to be more functional than structural. The structural aspect, such as the amount of hardware and the credentials of faculty, can be dealt with by pre-survey file reviews so as to reduce the time surveyors need to spend verifying these items on-site, enabling them to focus more on evaluating training plans.

In this category, standard 1.1.5 had the lowest pass rate in our pilot study. This standard states that all training plans for resident doctors and medical students should have a qualified program director. As will be further elaborated below, Taiwan started medical education and post graduate training reforms after the Severe Acute Respiratory Syndrome (SARS) outbreak. The DOH has set up training programs to train the trainers, i.e. the current faculty. As such, those programs can certify hospital staff to be various qualified training program directors. The reason for low compliance rate is that it was a fairly new initiative back in 2006 and a lot of hospitals had not sent out their staff to receive relevant trainings yet. Therefore, this standard remained unchanged after the pilot since it is necessary and we believe it will improve with time.

Category 2 not only includes training for medical students, interns and resident doctors, but also for paramedicals, including students in nursing, pharmacy, radiation technology and clinical laboratory students. During on-site surveys, students and resident doctors are interviewed to evaluate the performance of training plans. The new standards stress general medicine education, not specialty training. The reasoning is that evaluating specialty training programs should fall within the jurisdiction of specialty boards, and should not be in the hands of the TJCHA.

A new post-graduate general medicine training program for physicians [18] was started in 2003 right after SARS. Taiwanese health care professionals tried to create value from this drastic experience. One of the precious lessons we learned was the need to overhaul our resident training programs to instill a greater component of general medicine into over-specialized post-graduate training. In our medical education system, medical students spend their last of seven years of medical school as interns. After graduation, they enter resident training programs of their chosen specialties right away. This highly specialized approach does not serve the needs of the community well in a time of emergency epidemic. Therefore, the new program, called the PGY1 program, requires medical graduates to participate in a three-month general medicine training program during the first year of residency. The program includes fundamental courses and training in general internal medicine, general surgery and community medicine. The goal is to expand this training program to a one-year program in 2011. Since this was a new initiative when the study started, it was not included in the old teaching hospital accreditation standards. Later, this important change was incorporated into the survey process.

The other revision worth noting is that the lowest pass rate in this category was for standard 2.2.11. The original requirement was that the average overall pass rate in internal medicine, surgery, obstetrics and gynecology, and the pediatrics board exams for the past three years should be above 80%. This turned out to be a high bar because only around 50% of the pilot-tested hospitals could get beyond it. Therefore, it was subsequently revised downward to 75%.

For category 3, we stress the importance of promulgating incentives to encourage research and reward good results. These incentives cover not only physicians but also paramedicals.

In category 4, the new standards require hospitals to emphasize faculty development and put in place incentives for teaching so as to reward excellence. Hospitals are encouraged to have bylaws that encourage attending physicians to develop teaching careers.

For category 5, we stress the importance of vertical integration among different levels of hospitals, community outreach, and international exchanges and cooperation to provide teachers and students with diverse training and broaden their perspectives.

For category 6, administration, there are four major points: the operation of the administrative system, the operation of the medical education committee, budgeting and spending, and assessing teaching and research performance.

The language of the 95 standards may appear vague in terms of giving clear guidance about how to assess compliance. We have to point out that this study is not the end of the process. We still plan to write up scoring instructions for each standard so that surveyors can assess compliance systematically. Each standard will be given four scoring levels: A, B, C and D. A is the highest grade and C is a passing grade. For instance, if we simply read the language of standard 3.2.2, which states, "Physicians should have good research results," one may be really lost as to how to assess whether results are good or bad. The scoring instructions subsequently drafted for this standard instructs surveyors to look at the percentage of full time attending physicians who have published papers in peer reviewed academic journals in the past five years. The passing grade, i.e. C, requires 25% of the full time attending physicians to publish at least one academic paper as first or correspondence author under the hospital's name. B requires 50% and A 75%. The final results of teaching accreditation hinge on the sum of all the scores. However, the relative quality and impact of the publications have not been taken into account by this approach and this shortcoming also needs to be addressed in the future.

That said, there is no denying that assessment of good and bad is always value-laden and judgmental. Although we try to create objectivity with the help of scoring instructions, we cannot escape the fact that the determination is still made through the subjective observations of the surveyors. Therefore, the objectivity and consistency of surveyors have always been a problem for which they have often been criticized in the past. The TJCHA had also noticed this issue and took measures to ensure the quality of the accreditation process itself. Starting from 2004, as part of the hospital accreditation reform, it launched a new program for recruiting and training surveyors in order to ensure performance and objectivity [19]. In the new program, surveyors are constantly trained and evaluated.

Although the final decisions as to pass and fail are supposed to be made by the DOH, the cumulative scores of the surveyors for each survey in fact dictate the final results since TJCHA has transformed the survey findings numerical by the establishment of scoring instructions. The advantage is that the chances of arbitrary manipulation by government or TJCHA officials are minimized; whereas the disadvantage is that extra mechanisms have to be put in place in order to ensure the objectivity of the scoring processes. Therefore, normalization of survey scores is needed for each cohort of hospitals in the end. The way to go about it in Taiwan is by some sort of Delphi technique; after all the hospitals for that year have been surveyed, all the surveyors will gather for a final meeting in which average scores and their variances on all standards are disclosed to all surveyors. The surveyors individually can decide whether they will adjust their scorings after knowing the distribution of scores. The adjustment made in the final meeting will be the final score for the hospitals. The TJCHA does not apply any mathematical manipulation afterwards since the assessment of each surveyor has to be respected.

Our pilot results indicate that average compliance rates with the new standards are at least around 80% for each category, which is quite high for a new set of standards. Since the pilot surveys were conducted on volunteer institutions, we need to factor in the influence of selection bias. Larger hospitals are more likely to volunteer, even among the B level teaching hospitals, and are likely to be better poised to embrace the new standards. It is therefore not surprising that the compliance rates are high. However, the new standards will be applied across the board and to smaller teaching hospitals as well. The TJCHA needs to ensure that the new standards inspire hospitals to reach new heights and yet are realistic at the same time. We cannot set a bar that is too high and doomed to fail because the TJCHA will encounter insurmountable resistance from the hospitals. Furthermore, there is 20% room for improvement, which should pose a reasonable challenge for most hospitals.

All in all, in comparison with the old standard, our new teaching hospital accreditation standards focus more on evaluating process and outcome, and are in sync with the overall trend in hospital accreditation. Research excellence appears to be the most difficult area for hospitals to achieve. It is certainly true that good teachers are not necessarily good researchers. Nonetheless, researchers are expected to be capable of enhancing teaching quality in the hospital. Although we aim at strengthening both teaching and research, our standards also look at clinical care processes in order to understand the real operation of clinical teaching and the education quality that can be achieved through the provision of hospital services. This has also been recommended to avoid false filing of accreditation data by hospitals [20].

## Conclusions

Learning from past experience, the TJCHA spearheaded the hospital accreditation reform after its inauguration in the late 90 s. Then the SARS epidemic led to a new wave of changes in medical education and post-graduate medical training that needed to be incorporated into the teaching hospital accreditation. As such, after rigorous study and pilot testing, the new teaching hospital accreditation standards launched in the first decade of the 21^st ^century consist of six categories and 95 standards. Under the new standards, not only resident doctors' training but also medical students' training is evaluated. The scope of the standards have also been extended to encompass professionals in nursing, pharmacy, radiation technology and clinical laboratories. The new on-site survey focuses more on evaluating process and outcomes than the old standards. Pilot testing has proven new standards to be feasible and some pose reasonable challenges for hospitals to aspire to. Further assessment of the performance of the new set of standards is still needed once it is fully implemented.

## Competing interests

The authors declare that they have no competing interests.

## Authors' contributions

CIH and CMY participated in the study design, data collection and interpretation, performed the analyses and drafted the manuscript. CW supervised the study and participated in the study design, interpretation and drafting of the manuscript. All authors read and approved the final manuscript.

## Appendix. The new teaching hospital accreditation standards

### 1. Educational resources (20 items)

#### 1.1 Faculty

1.1.1 The chief of medical staff of DOH certified specialties should have the required qualifications.

1.1.2 The percentage of visiting staff who are DOH certified specialists must be appropriate.

1.1.3 The ratio of resident doctors to visiting staff who are DOH certified specialists should be reasonable.

1.1.4 The ratio of medical students to visiting staff who are DOH certified specialists should be reasonable.

1.1.5 All training plans of resident doctors and medical students should have a qualified program director.

1.1.6 All training plans for paramedical students should have a qualified program director.

1.1.7 The faculty to train paramedical students should have the required qualifications and the faculty-to-student ratio should be appropriate.

#### 1.2 Teaching and research facilities and equipment

1.2.1 Visiting staff should have their own offices.

1.2.2 The number of lecture halls, small group discussion rooms and conference rooms must be sufficient and there should also be sufficient computing facilities to look up information and conduct statistical analyses.

1.2.3 The hospital is equipped with internet educational equipment and is capable of tele-consultation.

1.2.4 The hospital can provide and produce pedagogical tools.

1.2.5 Research rooms are available and there is evidence of their research and teaching effectiveness.

#### 1.3 There are adequate and convenient mechanisms for looking up books and literature and an appropriate utilization rate

1.3.1 The hospital has purchased necessary books and journals, has proper management of the library, and has provided all the departments most updated library information.

1.3.2 Proper utilization rates of library collection.

1.3.3 Provide searching services.

#### 1.4 Clinical training environment

1.4.1 The hospital provides good outpatient training facilities, which includes learning convenience, health care quality, patient safety and privacy.

1.4.2 The hospital provides good emergency training facilities, which include learning convenience, health care quality, patient safety and privacy.

1.4.3 The hospital provides good inpatient training facilities, which includes learning convenience, health care quality, patient safety and privacy.

1.4.4 The hospital provides resident doctors and medical students with space and equipment needed for learning and training.

1.4.5 The hospital provides paramedicals and paramedical students with space and equipment needed for learning and training.

### 2. Teaching and training plans and outcomes (42 items)

#### 2.1 The execution and results of teaching and training plans for medical students

2.1.1 The objectives of the teaching and training plans for medical students are feasible and the content of the core curricula is appropriate.

2.1.2 The teaching contents for medical students are sufficient and there are complete records in their student profiles.

2.1.3 The numbers of patients cared for by medical students and those of their on-duty shifts are appropriate and suitable for learning, and there are proper mechanisms of instruction and supervision.

2.1.4 The quantity and quality of seminars are appropriate and the content is helpful for the learning of medical students.

2.1.5 Visiting staff hold teaching rounds regularly (including bedside teaching) and medical students attend teaching rounds every week.

2.1.6 The medical record writing (including admission notes, progress notes, discharge summaries and outpatient records) of students is complete and of appropriate quality.

2.1.7 Visiting staff should review and revise medical students' medical record writing when necessary.

2.1.8 There are comprehensive medical student teaching and learning performance assessments and two-way feedback mechanisms.

2.1.9 Proper safety and universal precaution training for medical students.

2.1.10 The learning outcomes of medical students are good.

#### 2.2 The execution and results of teaching and training plans for resident doctors

2.2.1 The objectives of the teaching and training plans for resident doctors must be feasible and the content of core curricula appropriate.

2.2.2 The hospital has passed the survey for post graduate general medicine training and continues to improve training quality.

2.2.3 The outpatient and inpatient teaching content (including bedside teaching) for resident doctors are sufficient and there are records in their learning profiles.

2.2.4 The numbers of patients cared for by resident doctors and those of their on-duty shifts are appropriate and suitable for learning, and there are proper mechanisms of instruction and supervision.

2.2.5 The quantity and quality of seminars are appropriate and the content is helpful for resident doctors.

2.2.6 Visiting staff hold teaching rounds (including bedside teaching) regularly and resident doctors attend teaching rounds every week.

2.2.7 The medical records (including admission notes, progress notes, discharge summaries and outpatient records) written by resident doctors are complete and have appropriate quality.

2.2.8 Visiting staff should countersign and revise, when necessary, resident doctors' medical record writing.

2.2.9 There are comprehensive resident teaching and learning performance assessments and two-way feedback mechanisms.

2.2.10 There is proper safety and universal precaution training for resident doctors.

2.2.11 The average overall pass rate for internal medicine, surgery, obstetrics and gynecology, and pediatrics board exams for the past 3 years is above 75%.

2.2.12 The results of interviewing and evaluating resident doctors are good.

#### 2.3 The execution and results of teaching and training plans for nursing students

2.3.1 The department of nursing has signed with nursing schools practicum contracts in which mutual responsibilities have been clearly stipulated.

2.3.2 The content and execution of the teaching plans for nursing students can match the needs of nursing students.

2.3.3 The hospital and the schools have regular review meetings.

2.3.4 The hospital and the schools jointly evaluate nursing students.

2.3.5 Interview nursing students to evaluate their performance and responses to clinical teachings.

#### 2.4 The execution and results of teaching and training plans for pharmaceutical students

2.4.1 The objectives of the teaching and training plans for pharmaceutical students are feasible and the content of core curricula is appropriate.

2.4.2 The required subjects for pharmaceutical students' practicum are clearly stated and there are proper mechanisms of instruction and supervision, and records in their learning profiles.

2.4.3 The quantity and quality of seminars are appropriate and the content is helpful for the learning of pharmaceutical students.

2.4.4 There should be performance assessments for the training of pharmaceutical students in order to understand whether training results fulfill the professional demand of pharmacists.

2.4.5 Two-way teaching and learning feedback mechanisms should be established.

#### 2.5 The execution and results of teaching and training plans for radiation technology students

2.5.1 The objectives of the teaching and training plans for radiation technology students are feasible and the content of the core curricula is appropriate.

2.5.2 The course content and teaching activities for radiation technology students are appropriate and academic seminars are regularly held.

2.5.3 The clinical teaching for radiation technology students is appropriate.

2.5.4 There are comprehensive teaching evaluations, and two-way teaching and learning feedback mechanisms for radiation technology students.

2.5.5 The learning outcomes of radiation technology students are good.

#### 2.6 The execution and results of teaching and training plans for clinical laboratory students

2.6.1 The objectives of the teaching and training plans for clinical laboratory students are feasible and the content of core curricula is appropriate.

2.6.2 The teaching content for clinical laboratory students are sufficient and there are complete records in their learning profiles. Academic seminars are regularly held and the content is helpful for the learning of students.

2.6.3 There are comprehensive teaching and learning evaluations, and two way teaching and learning feedback mechanisms for clinical laboratory students.

2.6.4 Proper safety and universal precaution trainings for clinical laboratory students.

2.6.5 The learning results of clinical laboratory students are good.

### 3. Research and results (9 items)

#### 3.1 There should be good research incentives

3.1.1 There should be bylaws and incentives to encourage physicians and paramedicals to participate in research and to reward good research, and all these arrangements should function properly.

3.1.2 Emphasize research ethics and verify the authenticity of research publications.

#### 3.2 The results of research project execution

3.2.1 There should be research projects that have been funded by the hospital or outside resources.

3.2.2 Physicians should have good research results.

3.2.3 The execution and results of paramedicals' research.

3.2.4 Research publications (including research projects and results) demonstrate inter-specialty integration.

#### 3.3 Human subject experimentation

3.3.1 There are comprehensive charters and operational procedures for the Institutional Review Board.

3.3.2 The obtaining of consent from human subjects and the protection of their rights are complete.

3.3.3 There are project review and supervision mechanisms.

### 4. Development of clinical faculty and continuing education(8 items)

#### 4.1 The execution and results of faculty development

4.1.1 There should be a faculty development system for physicians, which has been effectively operational.

4.1.2 There should be a faculty development system for paramedicals, which has been effectively operational.

4.1.3 Teaching incentives for full-time attending physicians should be clearly promulgated and executed fully so as to encourage them dedication to teaching.

4.1.4 Teaching incentives for paramedicals should be clearly promulgated and executed fully so as to encourage dedication to teaching activities.

#### 4.2 Continuing education

4.2.1 Continuing education in professional knowledge.

4.2.2 Continuing education of basic ability in general medicine (such as patient safety, health care quality, physician-patient communication, medical ethics and law, infection control, evidence-based medicine and medical record writing.)

4.2.3 Continuing education for the improvement of teaching.

4.2.4 Continuing education of paramedicals.

### 5. Academic exchanges and community education (8 items)

#### 5.1 Practical training collaboration domestically

5.1.1 There are substantive collaboration relationships among hospitals, and the content and interactions are good.

5.1.2 Training collaboration mechanisms have been established with other hospitals.

#### 5.2 Participate in international health, domestic and international medical aid and disaster relief

5.2.1 Participate in international health activities and establish collaborating mechanisms for teaching, advanced study and research.

5.2.2 Participate in domestic and international medical aid and disaster relief, and assist in establishing systems and personnel training.

#### 5.3 Continuing education of primary care physicians in the community

5.3.1 Organize various continuing medical education activities for primary care physicians.

5.3.2 Good exchange of information between the hospital and primary care physicians.

#### 5.4 Health education for community residents

5.4.1 Provide health information for community residents.

5.4.2 Change the health care seeking attitudes of the public.

### 6. Administration (8 items)

#### 6.1 The operation of administration

6.1.1 There is an administrative unit for medical education to ensure proper execution and resource allocation.

6.1.2 Every clinical department has proper numbers of supporting administrative staff for teaching and research.

#### 6.2 A medical education committee should be established

6.2.1 The organization, function and roles of the medical education committee.

6.2.2 The medical education committee has good interactions with all the other departments that have teaching responsibilities, helpful for the promotion of medical education.

#### 6.3 Funding for teaching, advanced study and research

6.3.1 There should be separate funding for teaching, research and advanced studies, which could be supported by budgeting and final accounting data. The proportions allocated to physicians and paramedicals should be appropriate.

6.3.2 The general medicine training funds supported by the DOH should be fully spent on the general medicine training program.

#### 6.4 Performance assessment of the execution of teaching and research and subsequent improvement

6.4.1 Each clinical department should have regular teaching performance assessments and should implement improvement measures.

6.4.2 Each clinical department should have regular performance assessments of and statistics on the execution of research projects.

## Pre-publication history

The pre-publication history for this paper can be accessed here:

http://www.biomedcentral.com/1472-6963/9/232/prepub
